# Increase in Gut Microbiota after Immune Suppression in Baculovirus-infected Larvae

**DOI:** 10.1371/journal.ppat.1003379

**Published:** 2013-05-23

**Authors:** Agata K. Jakubowska, Heiko Vogel, Salvador Herrero

**Affiliations:** 1 Department of Genetics, University of Valencia, Burjassot, Spain; 2 Department of Entomology, Max Planck Institute for Chemical Ecology, Jena, Germany; Stanford University, United States of America

## Abstract

*Spodoptera exigua* microarray was used to determine genes differentially expressed in *S. exigua* cells challenged with the species-specific baculovirus SeMNPV as well as with a generalist baculovirus, AcMNPV. Microarray results revealed that, in contrast to the host transcriptional shut-off that is expected during baculovirus infection, *S. exigua* cells showed a balanced number of up- and down-regulated genes during the first 36 hours following the infection. Many immune-related genes, including pattern recognition proteins, genes involved in signalling and immune pathways as well as immune effectors and genes coding for proteins involved in the melanization cascade were found to be down-regulated after baculovirus infection. The down-regulation of immune-related genes was confirmed in the larval gut. The expression of immune-related genes in the gut is known to affect the status of gut microorganisms, many of which are responsible for growth and development functions. We therefore asked whether the down-regulation that occurs after baculovirus infection affects the amount of gut microbiota. An increase in the gut bacterial load was observed and we hypothesize this to be as a consequence of viral infection. Subsequent experiments on virus performance in the presence and absence of gut microbiota revealed that gut bacteria enhanced baculovirus virulence, pathogenicity and dispersion. We discuss the host immune response processes and pathways affected by baculoviruses, as well as the role of gut microbiota in viral infection.

## Author Summary

Baculoviruses are large DNA viruses that infect invertebrates, mainly insects from the order Lepidoptera. They were first discovered to cause insects' epizootics and are now used worldwide as biocontrol agents. Extensive studies on baculovirus biology led to the discovery that they can serve as expression vectors in insect cells; recently they have also been considered as vectors for gene therapy. Baculovirus infection, like many other oral infections, starts with the invasion of the gut by viruses; the gut is a compartment colonized by a community of resident microbiota. In this study, we observed that baculovirus infection leads to the decreased expression of immune-related genes in a *Spodoptera exigua* cell culture as well as in the larval gut. Gut microbial loads were found to increase after baculovirus infection. A series of bioassays showed that the baculovirus performs better in the presence of microbiota in the gut. Our study shows that baculovirus infection leads to increase of microbiota loads in the gut and that the gut microbiota play a significant role in insect immunity and susceptibility to viral infections. These findings suggest that gut microbiota can be manipulated to improve biocontrol strategies that employ baculoviruses.

## Introduction

Baculoviruses are large DNA viruses that infect arthropods, mainly insects from the orders, Lepidoptera, Diptera and Hymenoptera. During their replication cycle, they produce two distinct morphological forms, the occlusion-derived virus that is responsible for transmission of the infection between insects and the budded virus that is responsible for spreading the infection within one insect host [1]. Baculoviruses have a long history of being used as microbial insecticides to control insect pest populations in forestry and agriculture [2]. With the development of insect cell cultures, it became possible to replicate baculoviruses *in vitro* and to investigate their replication cycle and the mechanisms of infection.

Baculovirus infection impacts a host in many ways. Not all tissues are equally affected and the infection process does not develop in the same way in different cell types. Typically, successfully infected host larvae die 3–10 days after the initial infection, and death is often accompanied by larval body liquefaction [3]. Although pathological effects are easily observed particularly in the later phase of infection, the processes that take place in the host in the early stages of infection are still not well understood.

Host-pathogen interaction can be viewed as an arms race between two enemies. The pathogen evolves to optimize host infection and its dispersion, and the insect attempts to improve protection against the pathogen. Insects' responses to virus infections include inducible reactions, such as immune responses as well as physical and chemical barriers that prevent viruses from establishing the infection [4]. Two types of immune responses are known, innate and adaptive. Whereas in vertebrates the immune system is composed of both, in invertebrates only innate responses are present. Innate responses include cellular and humoral response, which share the same signalling pathways [5]. Cellular responses refer to hemocyte-mediated responses like phagocytosis, nodulation and encapsulation. Humoral responses include identifying the invading microbes by pattern recognition proteins and subsequently synthesizing antimicrobial peptides (AMPs). The production of AMPs in insects is regulated by the signalling pathways Toll, Imd and JAK-STAT. Cellular and humoral responses depend on each other and interact in order to clear invading microbes from the organism. Both can lead to the activation of phenoloxidase cascade and subsequent melanization and to the production of reactive oxygen species [5].

Most of the studies on insect immune systems have been performed in *Drosophila* as a model organism. Research on *Drosophila* antiviral responses clearly indicate that antiviral responses differ from antimicrobial responses [6]. While in antibacterial and antifungal responses, Toll and Imd are the leading signalling pathways, in antiviral responses, the JAK-STAT pathway seems to play a main role [7]. Antiviral responses include the RNA interference (RNAi) machinery, which is especially active against RNA viruses; though recent studies show that it also contributes to fighting DNA viral infections [8], [9]. Although most of the studies of the insect immune system have been described in *Drosophila*, research on baculovirus infection of Lepidoptera suggest that they have a similar antiviral defence system. Host defense pathways implicated in resisting baculovirus infections include melanization and encapsulation [4]. Melanization depends on the prophenoloxidase (PPO) pathway, which, as in blood-clotting systems in vertebrates, leads to the isolation of the pathogen [10], [11]. For instance in *Helicoverpa* zea, which is semi-permissive to AcMNPV, the processes of melanization together with encapsulation were shown to be able to eliminate viral foci and attenuate disease progression [12]. Lately, it has also been suggested that factors other than only hemocytes' defensive responses may be involved in antiviral defense. Hirai et al. 2004 found that in a Bombycidae host, *Antheraea pernyi*, baculovirus infection induced the expression of hemolin, an insect immune protein, and that silencing of hemolin affected the progress of viral infection [13]. However, a more recent study was unable to detect changes in the expression of hemolin after infection with baculovirus in two species of Lepidoptera, suggesting that hemolin does not participate in the response to virus infection in all insects [14]. Recently, a number of transcriptional and proteomic studies of host gene expression have shown that immune-related genes are regulated after baculovirus infection [15]–[20], which may indicate that these genes are involved in the response to baculovirus.

After being ingested by the insect, baculoviruses reach the gut, one of the main physical barriers for the establishment of a systemic infection in the larvae. Immune aspects in insects were initially studied in the hemolymph and fat body, tissues traditionally attributed to immune responses. More recently and after recognizing the role that the gut plays in shaping immunity in mammals [21], research in insect immunity has also focused on the gut. Gut epithelial tissue has been found to express pattern recognition proteins as well as antimicrobial proteins, which indicates that the gut, in addition to the hemolymph, plays an important role in the immune responses of insects [22]–[24]. The immune system faces an enormous challenge especially in gut tissue as in addition to the invading pathogens, it must continuously confront microbiota present in the gut lumen. To date, little is known about the interaction between gut microbiota and baculovirus infection.

Gut microbiota are involved in various physiological functions, including digesting food and protecting against pathogen infections by occupying attachment sites, competing for nutrients and preventing colonization [25]. Recent studies also suggest that commensal microbiota present in the gut modulate insect immune responses [26], [27] and insect susceptibility to pathogens such as *Bacillus thuringeiensis* [28], [29]. In vertebrates, it is well recognized that gut commensal microbiota play a crucial role in the immune responses to pathogens and, to a great extent, shape gut immune system [21], [30], [31]. Studies show both promotion [32], [33] and suppression [34], [35] of pathogen invasion by gut microbiota in vertebrates. In invertebrate pathology, the effects of gut microbiota on pathogens have been also long studied, mainly in bacterial and fungal infections [36]–[39].

Numerous studies have shown that virulence and the pathogenicity of baculoviruses vary considerably depending on the food plant of the host [40]–[42]. Recently researchers have noted that plants that are consumed by insect larvae in large amounts shape gut commensal microbiota, and the observed differences in virulence depending on the food plant may be due to the differences in gut bacterial communities that in turn shape gut immunity. A new line of research, nutritional immunology, has emerged to study relationships between diet, immunity, and pathogenic processes and gut microbiota [43], [44]. Commensal bacteria communities in insects depend on diet and taxonomy [45]. Each insect species contains its particular microbiota, and the relationship of microbiota and the intestinal immune system is most often described as dynamic homeostasis. The mechanisms of maintaining gut homeostasis are obviously complex, taking into account continuously changing conditions such as food composition, pH, gut enzymes, fluctuating amounts of non-pathogenic bacteria, pathogenic bacteria and other pathogens, among other factors.

The expression of many host proteins is undoubtedly influenced by baculovirus infection. The knowledge of genes affected by virus infection may serve to improve baculoviruses as heterologous protein expression vectors [15] and as biocontrol agents [46]. Although previous studies report on changes in host gene expression after baculovirus infection [15]–[20], the consequences of such expression changes in insect physiology and resulting interactions with the virus have hardly been addressed [46]. In this study, we move beyond a descriptive nature of expression data to search for changes in the host gene expression which are reflected in host phenotype. We first present a comprehensive analysis of the host gene expression patterns of *Spodoptera exigua* cells infected with two baculoviruses, the highly specific *S. exigua* nucleopolyhedrovirus (SeMNPV) and the generalist *Autographa californica* nucleopolyhedrovirus (AcMNPV). A high throughput microarray analysis was performed in the Se301 cell line in order to take advantage of the homogeneous conditions that can be reached in cultured cells. Among the regulated genes after baculovirus infection, we observed the down-regulation of a large number of immune-related genes including antimicrobial peptides. After observing similar regulation by quantitative real-time polymerase-chain reaction (qRT-PCR) in larval guts, we investigated in more detail the impact of viral infection on gut microbiota, finding that baculovirus infection has an important effect and that intestinal microbiota play an important role in baculovirus pathogenesis.

## Results

### Microarray data analysis of *S. exigua* genes after SeMNPV and AcMNPV infection

**Figure 1:**
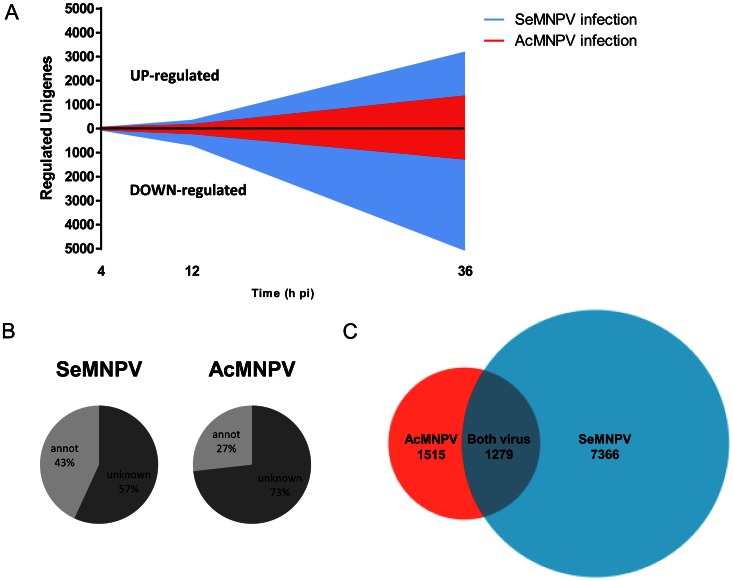
Gene expression patterns of *S.* exigua Se301 cells infected with SeMNPV and AcMNPV viruses, determined by microarray analysis. (A) Number of up- and down-regulated unigenes at 4, 12 and 36 h after treatment. (B) Percentage of baculovirus-regulated *S. exigua* unigenes that show homology to known or annotated genes available in public databases. Annot – known or annotated genes; Unknown – genes of unknown description. (C) Common and virus-specific unigenes regulated after treatment with SeMNPV and AcMNPV. In all panels only unigenes that show fold-changes higher than 2 (and p-value<0.05) are included.

Transcription profiles of close to 30,000 *S. exigua* unigenes were investigated in Se301 cells infected with the species-specific baculovirus SeMNPV and the non-specific, broad-range AcMNPV, in order to decipher host genes' responses and assess the extent of specific and general responses to viral infections. Only a very small number of genes were differentially regulated at 4 hours post infection (hpi) with the applied thresholds, 74 and 70 genes for SeMNPV and AcMNPV, respectively (Fig. 1A). At 12 hpi, the number of differentially regulated genes increased in cells infected with both SeMNPV and AcMNPV. However, the number of unigenes regulated after SeMNPV infection was three times higher than the number of genes regulated after AcMNPV infection (1000 and 344, respectively), suggesting that the gene transcriptional response to a species-specific virus is stronger and/or faster than the response to a non-specific virus. The highest number of differentially regulated unigenes was observed at 36 hpi, with an almost 4-fold difference between the response to SeMNPV and the response to AcMNPV. Over 8000 unigenes were differentially regulated after infection with SeMNPV, which exceeds 25% of all unigenes represented on the array. For AcMNPV infections, in contrast, only around 2600 unigenes (<10%) were differentially regulated. In neither case was the host gene expression shut-off observed, with 3177 up-regulated and 5053 down-regulated unigenes for SeMNPV and1341 up-regulated and 1250 down-regulated unigenes, for AcMNPV infections. For SeMNPV and AcMNPV infections, 43% and 27%, respectively, of differentially regulated unigenes showed homology to genes from public databases (Fig. 1B). Genes differentially regulated at 4, 12 and 36 h after SeMNPV and AcMNPV infections that have homology to genes present in public databases are listed in Supplementary Tables S2–S7.

### Common patterns in host genes' differential regulation after baculovirus infection

In order to select differentially responding unigenes common for both baculovirus infections, the lists of differentially regulated unigenes after SeMNPV and AcMNPV infections were compared. To reduce the possible influence of differences in the development of infection between both baculoviruses, the lists of unigenes from all three time points post-infection were pooled. In total, we found 1279 unigenes that were regulated after SeMNPV infection as well as after AcMNPV infection (Fig. 1C). Except for a few unigenes, most of common unigenes showed the same patterns of regulation, meaning that unigenes up-regulated in the host after AcMNPV infection were also up-regulated after SeMNPV infection, and unigenes down-regulated in the host after AcMNPV infection were also down-regulated after SeMNPV infection. Moreover, the regulation of individual unigenes, independent of the direction (up or down), was in general stronger in SeMNPV infected than in AcMNPV infected host cells. A large portion of unigenes differentially regulated by both baculovirus infections showed homology to genes from public databases (the top 10 differentially regulated annotated unigenes are presented in Table 1). A considerable portion of unigenes differentially regulated (14%) showed homology to genes involved in pattern recognition and immune responses, including antimicrobial effector proteins, such as attacin, gloverin and cecropin. Interestingly, most of the regulated immune-related unigenes were down-regulated in response to baculovirus infection. Among the most down-regulated unigenes, we found genes with homology to peptidoglycan recognition protein D (>30-fold, for SeMNPV infection), prophenoloxidase-activating enzyme 3 (>30-fold), beta-1,3-glucan recognition protein 2a (>15-fold), lysozyme-like protein 1 (>12-fold), pattern recognition serine proteinase (>10-fold), gloverin (>10-fold), attacin (>7-fold), immune-related Hdd23 from *Bombyx mori* (>6-fold), and cecropin (>5-fold). Moreover, unigenes with homology to ABC transporters, known to be affected in other DNA virus infection, such as herpes, HIV or hepatitis B virus (Hinoshita et al., 2001), were found to be down-regulated.

**Table 1:** Selected unigenes differentially regulated after baculovirus infection (common for SeMNPV and AcMNPV infections).

Target Name*	Unigene	Fold Se	p-value	Fold Ac	p-value	E-value	First Hit
C13333	SE_U17995	3733.51	2.60E-05	10.31	1.14E-03	1.94E-07	gi|221120185|ref|XP_002166754.1| PREDICTED: similar to predicted protein [Hydra magnipapillata]
C5429	SE_U09252	211.71	3.14E-04	4.05	1.05E-02	2.83E-01	gi|218505911|gb|ACK77613.1| FI09240p [Drosophila melanogaster]
C6166_anti	SE_U20521	111.33	8.23E-04	2.07	2.36E-02	5.78e-085	gi|91090366|ref|XP_968305.1| PREDICTED: similar to AGAP011964-PA [Tribolium castaneum]
C6530_anti	SE_U00849	86.08	6.30E-04	2.26	2.25E-02	0.087	gi|34099638|gb|AAQ57129.1| endonuclease and reverse transcriptase-like protein [Bombyx mori]
C2940	SE_U17987	56.02	3.84E-04	13.25	2.13E-03	4.90E-44	gi|266808630|gb|ACY78421.1| diapause bioclock protein-like protein [Helicoverpa armigera]
C6734	SE_U10661	49.04	2.66E-03	21.81	1.98E-02	6.27E-49	gi|66518243|ref|XP_396715.2| PREDICTED: similar to kelch-like 10 [Apis mellifera]
C16091	SE_U49713	45.49	3.08E-04	2.25	3.00E-02	1.04E-04	gi|170039890|ref|XP_001847752.1| tektin [Culex quinquefasciatus]
C17003	SE_U12244	44.11	9.98E-04	2.70	3.01E-02	3.60E-156	gi|1403598|gb|AAC47136.1| retinol dehydratase [Spodoptera frugiperda]
C19720	SE_U19342	41.77	2.93E-04	2.61	3.43E-02	1.13E-72	gi|237700829|gb|ACR15998.1| serine protease 39 [Mamestra configurata]
C14243	SE_U12365	34.66	1.75E-04	2.24	2.39E-02	3.42E-32	gi|170032190|ref|XP_001843965.1| sugar transporter [Culex quinquefasciatus]
C6473	SE_U18679	−58.12	1.27E-03	−2.81	7.55E-03	6.13E-38	gi|110826028|gb|ABH01082.1| esterase [Sesamia nonagrioides]
**C4522_anti**	**SE_U20576**	**−36.12**	**2.83E-04**	**−4.96**	**1.60E-03**	**1.51e-071**	**gi|154240658|dbj|BAF74637.1| peptidoglycan recognition protein-D [Samia cynthia ricini]**
**C12021**	**SE_U00298**	**−34.73**	**2.23E-05**	**−6.27**	**3.85E-05**	**3.25E-101**	**gi|83583697|gb|ABC24708.1| G protein-coupled receptor [Spodoptera frugiperda]**
**C18723_sense**	**SE_U28620**	**−30.22**	**1.34E-02**	**−4.78**	**1.44E-02**	**0.0139**	**gi|56718390|gb|AAW24481.1| prophenoloxidase activating enzyme 3 [Spodoptera litura]**
C8788	SE_U04807	−23.36	5.12E-04	−4.49	1.24E-02	1.07E-13	gi|110826028|gb|ABH01082.1| esterase [Sesamia nonagrioides]
**C4232**	**SE_U19694**	**−21.93**	**8.42E-03**	**−4.57**	**1.66E-02**	**0.00E+00**	**gi|56718390|gb|AAW24481.1| prophenoloxidase activating enzyme 3 [Spodoptera litura]**
C11961	SE_U12539	−21.88	7.15E-04	−2.80	9.79E-03	0.00E+00	gi|110826028|gb|ABH01082.1| esterase [Sesamia nonagrioides]
**C2788**	**SE_U20847**	**−17.90**	**2.91E-02**	**−3.16**	**1.13E-03**	**8.76E-159**	**gi|208972531|gb|ACI32826.1| beta-1,3-glucan recognition protein 2a [Helicoverpa armigera]**
**C13320**	**SE_U18431**	**−17.08**	**2.29E-03**	**−6.32**	**4.06E-04**	**1.72E-10**	**gi|242021463|ref|XP_002431164.1| class B secretin-like G-protein coupled receptor GPRmth4, putative [Pediculus humanus corporis]**
**C164_sense**	**SE_U17332**	**−16.74**	**6.17E-03**	**−4.02**	**2.60E-02**	**6.24e-038**	**gi|56718390|gb|AAW24481.1| prophenoloxidase activating enzyme 3 [Spodoptera litura]**

* Target name represents mean from two probes. Immune-related unigenes are in bold.

**Figure 2:**
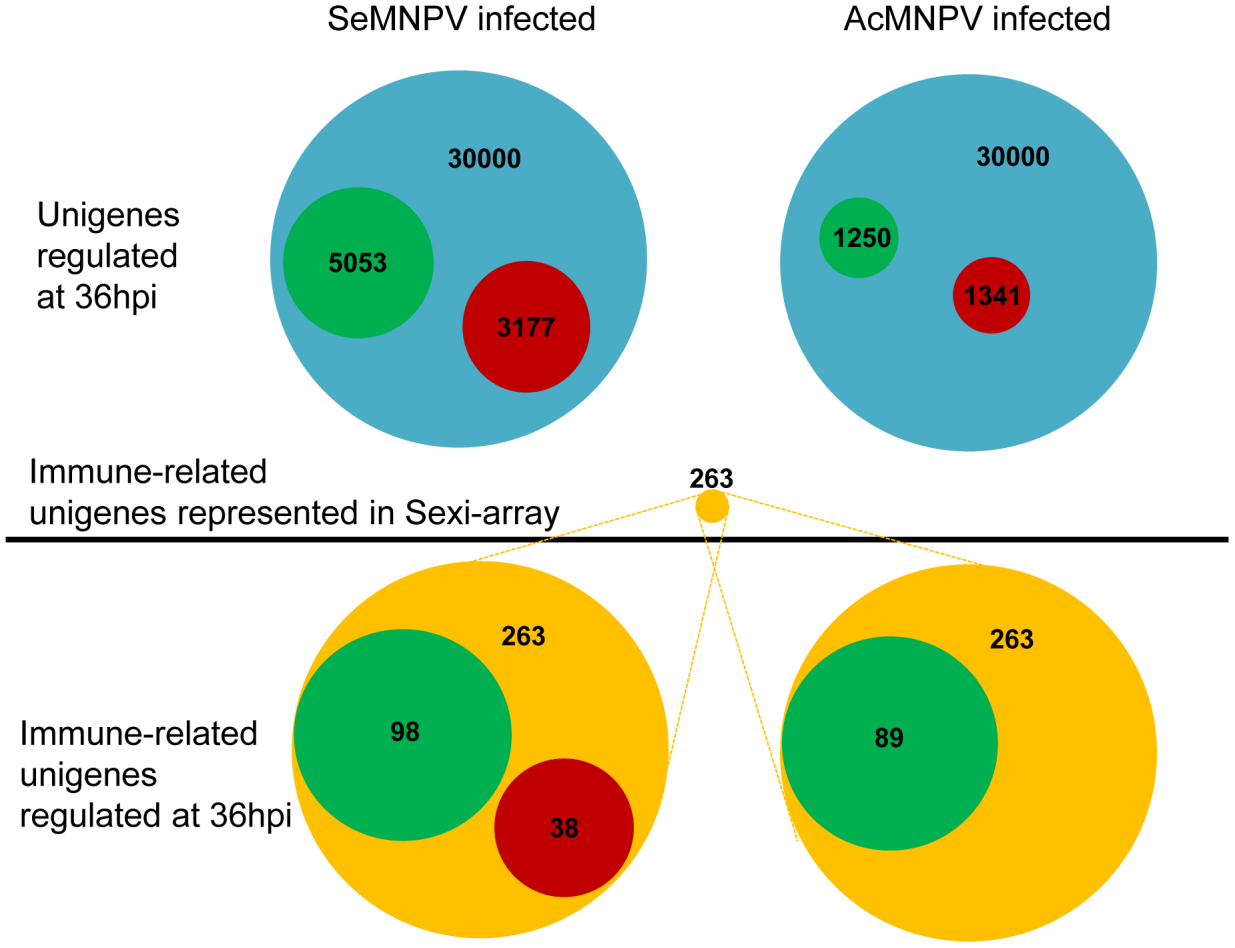
Proportion of unigenes and immune-related unigenes regulated in response to baculovirus infection at 36 hpi. Color code: blue – total number of unigenes represented in Sexi-array, green – down-regulated unigenes, red – up-regulated unigenes, yellow – total number of immune-related unigenes present in the Sexi-array. The size of the circle reflects the number of unigenes that it represents.

Given the high proportion of immune-related genes among genes regulated after baculovirus infection (by both viruses), an immune-related gene search was performed for each virus separately (Fig. 2). Immune-related unigenes were selected based on the relationship of their gene ontology terms with immune responses as well as their sequence homology to known immune-related genes. The immune-related genes that were identified constitute about 1% (263) of all unigenes represented on the Sexi-array. Many of these immune-related unigenes are regulated after SeMNPV and AcMNPV infection, with 136 unigenes and 89 unigenes showing expression differences, respectively. The majority of the immune-related unigenes were found to be down-regulated, with 72% (98 unigenes) and 100% (89 unigenes) down-regulated after SeMNPV and AcMNPV infections, respectively. The list of immune-related unigenes and their differential regulation fold-change values are presented in Supplementary Table S8.

### In vivo validation of selected genes by qRT-PCR

To validate the microarray results and extend them to in vivo conditions, 13 genes differentially regulated in Se301 after viral infection (several antimicrobial peptides, a pattern recognition protein, a phenoloxidase activating enzyme and a lectin) were selected; the response of these 13 genes to the viral infection was tested by qRT-PCR. Given that many immune-related genes are differentially regulated, which is generally attributed to insect defense against bacterial and fungal infections, we extended this study to two groups of larvae: one harboring laboratory levels of microbiota (referred to here as “with microbiota”) and another lacking microbiota (referred to here as “w/o microbiota”). Larvae “with microbiota” were reared on standard artificial diet [47] lacking antibiotics to maintain their natural laboratory microbiota levels, while larvae “w/o microbiota” were reared on the standard artificial diet supplemented with 0.2 g/l streptomycin; this supplement has previously been shown to remove a major part of culturable bacteria from the gut [28]. We also selected genes known to be involved in Toll and Imd pathways, and the JAK-STAT signalling pathway, the three main immune signalling pathways. Hypothesizing that gut tissue is where microbiota are located and interact with the insect, we searched for selected genes in larval guts using qRT-PCR.

**Figure 3:**
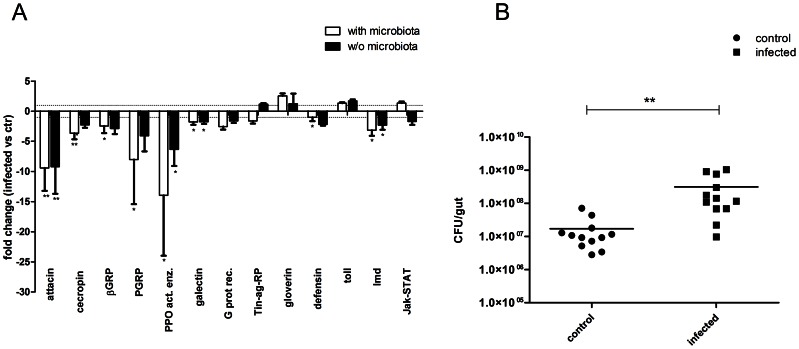
Immune-related genes expression and bacterial loads in the gut of infected *S. exigua* larvae. (A) *In vivo* validation of microarray data with qRT-PCR. Abbreviations: βGRP: beta-1,3-glucan recognition protein; PGRP: peptidoglycan recognition protein; PPO act.enz.: prophenoloxidase activating enzyme; G prot rec.: G protein receptor; Tin-ag-RP: tubulointerstitial nephritis antigen precursor. The expression of each gene in the gut of infected larvae was compared to its control in the gut of uninfected larvae. Each bar represents mean fold-change ±SD of three independent experiments, means were analyzed with t-tests, * p<0.05, ** p<0.01. (B) Bacterial loads in the midguts of *S. exigua* larvae with microbiota infected with SeMNPV in comparison to non-infected controls. Each point represents the number of bacteria per replicate (5 guts) and horizontal lines indicate the means; means were compared with the Mann Whitney test, **p<0.01.

Most of the selected unigenes showed the same patterns of regulation in infected *S. exigua* guts (Fig. 3A). In the genes studied, the biggest change was observed for the prophenoloxidase-activating enzyme. The peptidoglycan recognition protein (PGRP) and the β-glucan recognition protein (β-GRP) as well as antimicrobial effectors attacin and cecropin B also displayed clear down-regulation upon baculovirus exposure. Gloverin was down-regulated by infection in cell culture but no regulation in the larval gut tissue was observed. Among the three immune signalling pathway genes assayed in infected larvae, only the *Imd* gene was found to be down-regulated. Although the expression pattern was similar in larvae with and without microbiota, the level of regulation was in general stronger in larvae with microbiota.

### Increase in bacterial load in the gut of baculovirus-infected insects

A decrease in the expression level of immune-related transcripts, including immune response effectors such as antimicrobial peptides known to target bacteria, suggested that viral infection could be impacting the intestinal microbiota homeostasis. To test this hypothesis, *S. exigua* larvae were infected with SeMNPV, and culturable bacterial loads were determined and compared to the non-infected controls. A diet without antibiotics was used in this experiment to avoid the effects of antibiotics on viral infection. A significant increase (18.2-fold) in the intestinal bacterial loads was observed in the larvae infected with SeMNPV, in comparison to non-infected controls (Fig. 3B). Two types of bacterial colonies were observed among the culturable bacteria from the gut of *S. exigua*. 16S rRNA typing of these colonies revealed that they belong to the *Enterococcus* and *Enterobacter* genera, with approximately 100-fold higher counts for *Enterococcus*. In order to discount the possibility that bacteria could be leaking from the gut into the hemolymph due to the viral infection and lead to the observed differences in gene expression, we have also tested the bacteria in the hemolymph of our larval samples (data not shown). No significant differences in bacterial counts were observed between infected and control larvae.

### Effects of gut microbiota on SeMNPV pathogenesis in *S. exigua* larvae

We tested whether the decrease in the level of immune-related transcripts as well as the increase in the microbial loads in infected larvae has an impact on baculovirus pathogenesis. In other words, we aimed to determine if the down-regulation of immune-related genes in the intestine provides an advantage to the host or to the pathogen. For that purpose, *S. exigua* larvae with and without microbiota were infected with SeMNPV, and the infection process was monitored in terms of mortality and time to death, viral occlusion bodies (OBs) production and liquefaction.

**Figure 4:**
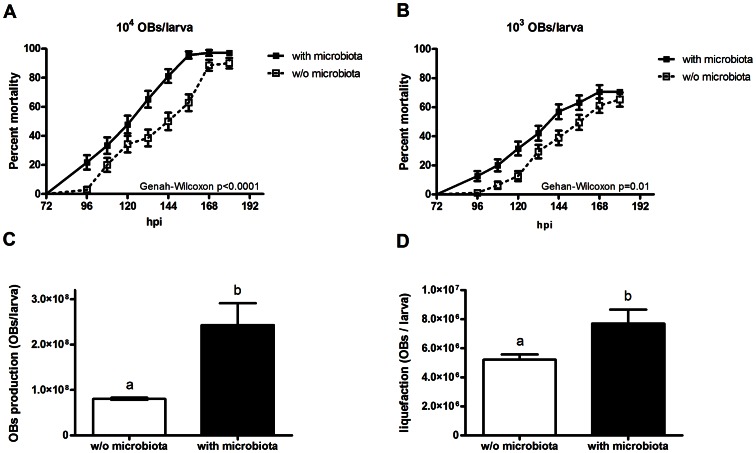
Effects of gut microbiota on SeMNPV pathogenesis in *S. exigua* larvae. (A) Survival at a dose 10^3^ OBs/larva (B) Survival at a dose 10^4^ OBs/larva (C) Virus production and (D) Liquefaction of larvae reared on diet containing streptomycin (0.2 g/l) (w/o microbiota) and diet without antibiotic (with microbiota). Survival curves were plotted using the Kaplan-Meier method. Statistical significance was determined using Gehan-Wilcoxon analysis. The survival curves of larvae with and without microbiota are significantly different as determined by Gehan-Wilcoxon analysis. P-values are reported. In (C) and (D) bars denote mean ±SD. Means were analyzed with t-tests; different letters denote significant difference with p<0.05. N = 3.

Time to death, as a measure of virulence, was assessed for larvae with and without microbiota by comparing their survival plots using the Kaplan-Meier method. Larvae with microbiota died faster (median survival 132 h) than larvae without microbiota (median survival 150 h), when infected with 10^4^ OBs/larva (p<0.0001, Gehan-Wilcoxon test). For this dose the mortality was 99% among the larvae with microbiota and 89% among the larvae without microbiota (Fig. 4A). When infected with a lower dose, 10^3^ OBs/larva, a significant difference in survival time was again observed between the larvae with and without microbiota (p = 0.0138, Gehan-Wilcoxon test). The mortality was 71% for the larvae with microbiota and 65%for the larvae without microbiota (Fig. 4B).

OBs' productivity and liquefaction capability were also calculated for both larvae harboring gut microbiota and those lacking gut microbiota. Larvae with microbiota produced 2.8-fold (t-test, p<0.05, t = 5.8, df = 4) more OBs than did larvae without microbiota (Fig. 4C). Similarly, the release of OBs to the environment was higher (1.5-fold, t-test, p<0.05, t = 4.3, df = 4) for larvae with gut microbiota in comparison to larvae without gut microbiota (Fig. 4D).

## Discussion

The species-specific *S. exigua* microarray (Sexi-array) was developed in this study in order to elucidate sets of genes that are regulated after baculovirus infection. The microarray data from cells infected with two different nucleopolyhedroviruses, SeMNPV and AcMNPV, showed two main trends that occur in the cells after infection. The first was the lack of a general down-regulation of the host gene expression (host gene shut-off) that, according to published data, was expected to take place within 36 hpi. The second was the down-regulation of genes involved in pattern recognition and immune responses.

The present study extends the previous works in other lepidopteran species (both *in vivo* and *in vitro*) on the differential expression of host genes after infection with baculoviruses. Host gene shut-off at late times post-infection was commonly observed; however, the responses seem to be specific for the insect species, the infective virus and even for the studied tissue. The first report on how baculoviruses regulate hosts' RNA levels [48] showed that the expression of three conserved *Spodoptera frugiperda* genes, actin, hsp70 and histone, decreased dramatically between 12 and 18 hpi in Sf21 cells infected with AcMNPV. This early study has been recently complemented by a microarray-based comprehensive analysis of host genes in AcMNPV-infected Sf21 cells [15]. Out of 42,000 probes included in the array, 70% were differentially regulated, with nearly all of the genes being down-regulated. Earlier studies using the differential display approach [20] also showed a global down-regulation of Sf9 genes after AcMNPV infection. Our results show that baculovirus-infected Se301 cells did not entirely follow this pattern. We did observe that there were more genes down-regulated than up-regulated as the duration of infection increased; nevertheless, even at 36 hpi many genes were up-regulated. Similarly, and although tested at earlier times post-infection, no global down-regulation of host genes has been reported in *Helicoverpa zea* cells HzAM1 infected with *Helicoverpa armigera* single nucleopolyhedrovirus (HearNPV) [49] and after injection of *S. exigua* larvae with AcMNPV [16], at 18 and 12 hpi, respectively. These results suggest that the extent of host gene shut-off strongly depends on the specific host-virus interaction.

Insect immune responses were originally thought to be a response to bacteria and fungi [50], although recent studies have also shown that innate immunity may be involved in viral infections [51]. In our study, gene expression analysis revealed that immune-related genes are down-regulated after the infection of *S. exigua* cells with two different baculoviruses. We also observed this effect in the gut tissue of *S. exigua* larvae infected with SeMNPV. Confirming our results, Choi et al. [16] recently described in *S. exigua* (entire larvae) the down-regulation of immune-related genes 12 h after the injection of AcMNPV in the larval hemolymph. However, few studies focusing on the insect hemocytes or fat body have reported the induction of immune-related genes after baculovirus infection. Although a general immune induction due to the injection of foreign material could occur, Moreno-Habel et al. and Wang et al. [17], [52] found that gloverins were induced in the hemocytes of *Trichoplusia ni* and *H. armigera* larvae injected with AcMNPV. In *in vitro* studies, preincubating AcMNPV virions with Sf9 supernatant containing recombinant gloverins decreased viral infectivity, which led the authors to conclude that gloverin has antiviral activity in AcMNPV-infected Sf9 cells [52] and to speculate that the membrane-bound gloverin could act as a virus entry molecule. Hirai et al. [13] found hemolin had been induced in the pupal fat body after baculovirus injection. Considering the possible antiviral activity that has been associated with some immune-related proteins, the down-regulation of immune-related proteins observed in the gut could be a mechanism the virus relies on to improve its ability for a successful infection. Viruses, including baculoviruses, have been reported to manipulate host physiology for their own benefit [53]–[55].

On the other hand, the down-regulation of immune-related genes in the gut was expected to have an impact on gut microbiota. Evaluating the influence of baculovirus infection on bacterial loads in the gut, we found that baculovirus infection led to an increase in gut microbiota; this increase may be related to a decrease in antibacterial activity due to the observed down-regulation of immune-related genes. As speculated above, the down-regulation of immune-related genes in the gut as a result of virus infection may directly benefit the virus, but the role of the third participant in the system, gut microbiota, must also be accounted for. After observing that gut bacterial loads change after baculovirus infections, we measured how the baculovirus infection process proceeds in the presence and absence of gut microbiota. Four parameters of viral fitness were measured in larvae with and without gut microbiota after they were infected with a baculovirus: pathogenicity (measured as larval mortality), virulence (measured as time to death), viral multiplication, and viral dispersion (measured as OBs' productivity and liquefaction, respectively). For all tested parameters, the virus benefited from the presence of gut microbiota. Are increased loads of gut bacteria a side-effect of virus infection? Or are we observing a more complex mechanism according to which the down-regulation of immune defenses against gut bacteria is orchestrated by the virus for its own benefit? Likewise, if bacterial numbers are increasing, could a virus infection also be directly beneficial to the gut microbiota? These questions point to directions for future research. What remains clear is that the role of gut microbiota cannot be ignored when studying virus infection.

Assuming that a virus influences insect immunity for its own benefit, a question arises: what is the mechanism by which baculoviruses manipulate host immune responses? Recent studies have shown that baculoviruses encode microRNAs that target both viral and host genes [56]. In the *Bombyx mori* NPV genome, several microRNAs were computationally predicted [57], and the effect of one of them in increasing the viral load has been recently confirmed [58]. Among the targets predicted were host defense elements such as prophenoloxidase and hemolin, which suggests that virus miRNAs constitute a tool with which host immune systems can be manipulated. More genetic information from *S. exigua* would facilitate the identification of baculovirus-encoded miRNAs that may be targeting some of the down-regulated immune-related genes.

We also explored the regulation of the main immune-related pathways in insects, namely Toll, IMD and JAK-STAT, by analyzing the expression of key genes in these pathways. In our study, neither the Toll-like receptor nor the transcription factor STAT was found to be regulated. In contrast, the *Imd* gene was found to be regulated in both *in vitro* and *in vivo* experiments. In both cases, the decline in the expression of *Imd* was observed. We speculate that the IMD pathway may be involved in the response to baculovirus infection and that baculoviruses may manipulate microbiota loads by down-regulating this specific signalling pathway. Likewise, the Imd pathway has also been shown to be involved in the viral response to RNA viruses (Alfaviruses) [59].

Another question that remains to be elucidated is related to the mechanisms underlying the enhancement of baculovirus pathogenicity by gut microbiota. Bacterial colonization and pathogenicity relies on the synthesis and secretion of virulence factors. Virulence factors enable microorganisms to establish themselves on or within a host of a particular species and enhance their potential to cause disease. These virulence mechanisms are also relevant for commensal microbiota, which are not lacking virulence factors and, once they invade hemocoel, can ultimately lead to insect death [60]. Bacterial virulence factors might contribute to the success of viral infections. For example, various types of bacteria produce chitin-degrading enzymes [61], [62]. In our case, chitin degradation by gut bacteria may have contributed to the establishment of the viral infection. Baculoviruses also produce chitinase in order to degrade host tissues and facilitate viral dispersion [63]. Therefore, chitin-degrading factors from both bacteria and viruses, may have acted simultaneously, increasing the viral dispersion observed in our study. Both in a previous study and here we observed that laboratory culturable microbiota in our colonies of *S. exigua* are mainly composed of species of the genus *Enterococcus* [28] and *Enterobacter*. Virulence factors from *Enterococcus spp*. include several exoenzymes such as gelatinases, hyaluronidase, and serine proteases [64], [65]. Those enzymes may have contributed to the spread of the virus by degrading the extracellular matrix. Additional studies should be undertaken in order to discover bacterial elements that contribute to baculovirus pathogenicity.

Microbiota have to be considered when studying host-pathogen interactions especially for orally infecting pathogens [66]. In the case of viral pathogens, the influence of microbiota can be either protective [34], [55] or harmful [32], [33], [67]. Our study shows that the baculovirus infection process increases gut microbiota loads in the insect host and that such an increase enhances baculovirus fitness. The gut architecture of invertebrates should be taken into account when studying virus-host interactions and the system should be perceived as composed of four elements: virus-microbiota-host-diet. The challenge arises when we ask how we can manipulate the gut microbiota to optimize the use of baculoviruses for the biocontrol of insect pests. Manipulating symbiotic bacteria has been shown to reduce insect-vectored human diseases, such as dengue virus, after antiviral protection was associated with the presence of *Wolbachia* spp [68]. Interestingly, in the lepidopteran *S. exempta, Wolbachia* spp. were found to increase the host's susceptibility to baculovirus infection. An approach based on exploiting bacterial endosymbionts for more effective pest control has been proposed [69]. Similarly, the manipulation of gut microbiota could be considered in biocontrol strategies in order to increase pest susceptibility to baculoviruses as well as a way to reduce viral susceptibility in other organisms.

## Materials and Methods

### Insects, virus and cells

Baculovirus-free *S. exigua* larvae were kindly provided by Primitivo Caballero from the Public University of Navarra, Pamplona, Spain. The colony was regularly checked for the presence of baculovirus infection by qRT-PCR and proved to be negative for SeMNPV. The insects were reared at 25°C, relative humidity of 70% and a photoperiod of 16 h∶8 h (light∶dark), on artificial diet [47]. The viruses used in the experiments were SeMNPV (SP2 and Ox4 isolates) [70], [71] provided by Primitivo Caballero from the Public University of Navarra, Pamplona, Spain and AcMNPV (C6 isolate) [72]. *S. exigua* Se301 cells were maintained in HyQ-SFX insect cell culture medium supplemented with 5% FBS at 25°C, according to standard cell maintenance procedures.

### Preparation of the virus for cell culture infections

*S. exigua* L4 larvae were orally infected with SeMNPV and AcMNPV virus at doses of 10^4^OBs/larvae and 10^5^OBs/larvae, respectively. Larvae were offered a suspension of OBs added to a small piece of diet. Larvae that ingested diet plugs containing virus suspension within approximately 2 h were provided fresh diet and considered in the assay. Four days post-infection (dpi), the hemolymph was collected from infected larvae by cutting prolegs. The hemolymph was filtered by 0.45 µm filters, and Se301 cells were infected at a high multiplicity of infection (MOI) with the budded viruses (BVs) present in the hemolymph for four hours. Five days post infection (dpi), the BV-containing supernatant was collected from the cells and filtered as before; virus titers were then calculated in Se301 cells using an end point dilution assay [73]. Viruses prepared in such a way were subsequently used to infect Se301 cells. A control sample was prepared in the same way, although L4 *S. exigua* larvae were mock-infected.

### Microarray design and synthesis

A 44K Agilent oligonucleotide chip was designed to include more than 40,000 probes from close to 30,000 *S. exigua* unigenes. The sequences of *S. exigua* were obtained from a *S. exigua* transcriptome sequencing project described elsewhere [74]. The *S. exigua* Agilent custom microarray (Sexi-array) was designed based on obtained sequences using eArray application from Agilent. Most of the unigenes were represented by two 60-mer oligonucleotide probes, designed to target different fragments of each unigene.

### RNA extraction, labelling and hybridization

Se301 cells were infected by incubation with SeMNPV and AcMNPV at a MOI of 5 for four hours. At 4, 12 and 36 hours post-infection (hpi), cells were collected and total RNA was extracted using RNAzol reagent (Molecular Research Center, Inc., Cincinnati, OH), according to the manufacturer's protocol. To further purify the RNA, an RNAeasy Kit (Qiagen, Hilden) was used following the manufacturer's protocol. The quality of RNA was assessed by an Agilent 2100 Bioanalyzer using the EukaryoteTotal RNA Nano protocol. Three independent biological replicates were performed for each treatment and each time point.

Agilent One-Color Spike-in Mix was added and 600 ng of total RNA was used for cRNA (complementary RNA) synthesis. 1.65 µg of the resultant cRNA was fluorescently labelled with cyanine-3-CTP, fragmented and hybridized to *S. exigua* microarray slides following the One-Color Microarray-Based Gene Expression Analysis (Quick-Amp labelling) protocol from Agilent. *S. exigua* microarrays were scanned using a G2505B Agilent scanner and data were extracted using Agilent Feature Extraction 9.5.1 software. Before data analysis, hybridization quality control reports were verified as correct. RNA labelling and hybridization as well as array scanning and data extraction were performed by the Microarray Analysis Service of Principe Felipe Research Centre (CIPF), Valencia, Spain.

### Microarray data analysis

Data analysis was performed using free Babelomics 4.3 software (http://babelomics.bioinfo.cipf.es/) [75]. First, arrays were normalized using RNA spike-in probe data and quantile normalization methods. Normalized arrays of the samples treated with the virus were compared to controls at 4, 12 and 36 h after treatment and expressed as a fold-change in the gene expression. The thresholds of fold-change ≥2 and p-value<0.05 were applied. T-tests were used to compare gene expression between the arrays, both those infected with virus and the control, for each time point and each treatment.

### *In vivo* data validation by quantitative qRT-PCR

*S. exigua* larvae reared on two types of diets were used in the *in vivo* validation experiment. One group was reared on the standard diet lacking antibiotics to maintain natural laboratory levels of microbiota in their guts. The other group was reared on the standard diet supplemented with streptomycin (0.2 g/l) to deplete intestinal microbes. Streptomycin was previously shown to remove a major part of culturable bacteria from the larval guts [28].

*S. exigua* L4 larvae from both types of diet were individually challenged with SeMNPV at a dose of 10^4^ OBs/larvae by adding virus suspension to a diet plug. Control larvae were fed with water. Six larvae were used per each treatment and condition, and the experiment was performed three times. Guts from six larvae were collected at 72 hpi and pooled, and total RNA was extracted using RNAzol reagent. 1 µg of RNA was used for cDNA synthesis. RNA was first treated with DNase I (Invitrogen) and subsequently reverse-transcribed to cDNA using oligo-d(T) primer and SuperScript II Reverse Transcriptase (Invitrogen) according to the manufacturer's protocol. Thirteen genes were selected for validation with qRT-PCR, including several antimicrobial peptides and some immune-related genes. Additionally, as representatives of the three main signalling pathways in insect immunity, *S. exigua* homologs for Toll, Imd, and JAK-STAT pathway genes [74] were also included in the analysis. An ATP synthase gene served as a reference gene for qRT-PCR data normalization. Selected gene primer sets were designed using Prime Express software from Applied Biosystems (Supplementary Table S1). qRT-PCR was carried out in the ABI PRISM 7000 from Applied Biosystems. All reactions were performed using Power SYBR Green PCR Master Mix (Applied Biosystems) in a total reaction volume of 20 µl. Four µl of the 1∶5 diluted cDNA templates was added to each reaction. Forward and reverse primers were added to a final concentration of 300 pM. Expression ratios were calculated based on the formula (Ct_gene of interest, treated_−Ct_reference gene, treated_)/(Ct_gene of interest, control_−Ct_reference gene, control_), 2^−ΔΔCt^, which assumes 100% efficiency of all amplification reactions. The standard deviation of the ΔCt values of treated and control samples was calculated as (s_1_^2^+s_2_^2^)^1/2^, where s_1_ is a standard deviation of gene of interest Ct and s_2_ is a standard deviation of reference gene Ct. The standard deviation of ΔCt was then incorporated into the fold-difference calculation, after Applied Biosystems “Guide to Performing Relative Quantitation of Gene Expression Using Real-Time Quantitative PCR”.

### Bacterial loads in the larvae infected with baculovirus

*S. exigua* L4 larvae were infected orally with SeMNPV at a dose of 10^4^ OBs/larva. Control larvae were mock-infected. At 4 dpi, larvae were dissected and their guts isolated. For each sample, the guts of five larvae were pooled. The experiment was performed 3 times in quadruplicate. 1 ml of 0.1% Triton X-100 in PBS was added to each sample and the samples were vortexed vigorously. 100 µl of serial dilutions was plated on LB-agar plates and grown o/n at 37°C. Colony-forming units (CFUs) were counted and calculated as CFU/ml.

Bacterial loads were also counted in the hemolymph of infected and control larvae. Larvae infected as above were bled at 4 dpi and 100 µl of serial dilutions of hemolymph were plated on LB-agar plates and grown o/n at 37°C. CFUs were counted and calculated as CFU/ml.

### Bioassays

#### Mortality, mean time to death, OBs productivity and larval liquefaction

*S. exigua* L4 larvae reared on the two types of diet described before were individually infected *per os* with SeMNPV at two doses, 10^3^ and 10^4^ OBs/larva, by adding virus suspension to a diet plug. Mortality was recorded every 12 h until the death or pupation of all the larvae. Sixteen to thirty-two larvae were used for each treatment and condition, and the experiment was performed three times. Mortality was expressed in the percentage of dead larvae and time to death was assessed by comparing the survival curves of larvae with and without microbiota, using the Kaplan-Meier method (GraphPad Prism version 5). Statistical significance was determined using log-rank analysis (Mantel-Cox test) and Gehan-Breslow_Wilcoxon test, and both p-values reported.

To assess OB productivity, *S. exigua* L4 larvae were infected with SeMNPV at a dose of 10^4^ OBs/larvae and reared individually. To prevent the influence of the different times until death of the individual larvae, only larvae that died at the same time post-infection were included in this experiment. Larvae 5 dpi were separated from the diet and larvae that died within 12 h after separation were collected and pooled for OB isolation and quantification. OBs from each treatment were purified from the larval tissues as follows: Dead larvae were homogenized in 0.1% SDS in PBS and filtered through three layers of cheesecloth. The filtrate was clarified by centrifugation at 1000 g for 3 min to remove large debris and subsequently centrifuged at 13,000 g for 15 min. The resulting pellet was resuspended in water and counted. OBs from each treatment were pooled and counted using an improved Neubauer chamber. Ten larvae were used for each treatment and three biological replicates were performed.

Larvae liquefaction was assessed in a separate experiment. *S. exigua* L4 larvae were individually infected with SeMNPV at a dose of 10^4^ OBs/larva. As before, larvae 5 dpi were separated from the diet, and larvae that died within 12 h after separation were used in the experiment. Tubes with the dead larvae were briefly vortexed, and 100 µl of PBS was added to each tube. OB-containing PBS (50 µl) was collected and OBs were counted as described before. Ten larvae were used for each treatment and the experiment was performed three times.

### Statistical analysis

Microarray data analyses were performed in Babelomics as described above. qRT-PCR data were analyzed according to Applied Bioscience guide. The rest of the bioassays were analyzed using GraphPad Prism version 5. Bacteria loads in the gut counts were analyzed using the non-parametric Mann Whitney test and virus performance assays were analyzed with unpaired t-tests. Data are reported as means ± standard deviation (SD). Differences between compared groups were considered significant when p<0.05.

## Supporting Information

Table S1 Sequences of the primers used for qRT-PCR.
(DOCX)

Table S2 Unigenes differentially regulated at 4 hpi after AcMNPV infection showing homology to genes available in public sequence databases.
(XLSX)

Table S3 Unigenes differentially regulated at 4 hpi after SeMNPV infection showing homology to genes available in public sequence databases.
(XLSX)

Table S4 Unigenes differentially regulated at 12 hpi after AcMNPV infection showing homology to genes available in public sequence databases.
(XLSX)

Table S5 Unigenes differentially regulated at 12 hpi after SeMNPV infection showing homology to genes available in public sequence databases.
(XLSX)

Table S6 Unigenes differentially regulated at 36 hpi after AcMNPV infection showing homology to genes available in public sequence databases.
(XLSX)

Table S7 Unigenes differentially regulated at 36 hpi after SeMNPV infection showing homology to genes available in public sequence databases.
(XLSX)

Table S8 Unigenes showing homology to immune-related gene.
(XLSX)
