# Supplementary material for: Increase in Gut Microbiota after Immune Suppression in Baculovirus-infected Larvae
Source: PLoS Pathog. 2013 May 23;9(5):e1003379. doi: 10.1371/journal.ppat.1003379 (PMC3662647; doi:10.1371/journal.ppat.1003379)
Supplement: Table S1 — Sequences of the primers used for qRT-PCR. (DOCX) [file ppat.1003379.s001.docx]

Table S1 Sequences of the primers used for qRT-PCR

| **Target Gene** | **Primer Name** | **Sequence 5´ → 3´** |
| --- | --- | --- |
| Attacin | A146-c2524-attacin-qF | TGCCAATCTGTTCCACAATCA |
|  | A147-c2524-attacin-qR | TGAGGGATGGTGGGCATATT |
| Cecropin B | A148-c2678-cecrb-qF | TCGCTTTGAGCGCTGTCA |
|  | A149-c2678-cecrb-qR | CGACCCACCTTTTCAATTTTCT |
| Beta-glucan recognition protein | A144-c1305-beta-glu-qF | AATTGGAAGCCATCTATCCTAAAGG |
|  | A145-c1305-beta-glu-qR | TGAGGTTTCCGTGGAATGC |
| Peptidoglycan recogniztion protein | A152-c5919-pept-rec-qF | GTAGTACCGGAGTGTGTTAGTGATGAG |
|  | A153-c5919-pept-rec-qR | TTGTCCTATATCAGTGAATCCACGTT |
| Prophenoloxidase activating enzyme | A150-c4232-PPOact-qF | AGCTGTGCGGCCCAGAT |
|  | A151-c4232-PPOact-qR | TCGACACCGCAACATTCACT |
| Galectin | A154-c7762-galectin-qF | GCCCCTGAGTATTGGATCACA |
|  | A155-c7762-galectin-qR | GCAAACCAGGGCAAATCGT |
| G-protein receptor | A156-c12021-Gprot-rec-qF | GGCCGTCAGTGTGAAGAATATTAAGT |
|  | A157-c12021-Gprot-rec-qR | ACGGGAACAGCAAATTGTTGT |
| TIN-ag-RP | A158-c15104-tubulo-qF | CGATGACTGTTGCCCAGACTAC |
|  | A159-c15104-tubulo-qR | TGCAGCCCATGGTGTTATATTC |
| Gloverin | G178-gloverin-qF | GAAATGCTGGAAGAGGCAAG |
|  | G179-gloverin-qR | ACCTCGGCCGAATAAAGAGT |
| Defensin | G174-defensin-qF | GTTTGCCAGGAGCATTGTCT |
|  | G175- defensin -qR | TAACGCAGATGCCGTAAGTG |
| Toll receptor | A162-15642-toll-qF | TTCTTTAGTCTTTTCCAGAACATTGG |
|  | A163-15642-toll-qR | ACCTGATGCTGACAAAGACCTACA |
| Imd | A176-U19714-Imd-Se-qF | GCTCCAAGGCCATCTACAGAGA |
|  | A177-U19714-Imd-Se-qR | TCCTGATCTTCATTTTGATCTTGATT |
| JAK-STAT | A178-U12797-JAK-qF | CGCCCTTACAGGATCATCTCA |
|  | A179-U12797-JAK-qR | AGGCCGGATTCTAGGAGCTT |
| ATP synthase | G56-ATPsynthase-Forw | GTTGCTGGTCTGGTGGGATT |
|  | G57-ATPsynthase-Rev | AGGCCTCAGACACCATTGAAA |
